# Using Routine Data to Improve Lesbian, Gay, Bisexual, and Transgender Health

**DOI:** 10.2196/53311

**Published:** 2024-05-01

**Authors:** Catherine L Saunders

**Affiliations:** 1 Department of Psychiatry University of Cambridge Cambridge United Kingdom

**Keywords:** lesbian, gay, bisexual, trans, LGBTQ+, routine data, England, United Kingdom, health, viewpoint, sexual orientation, health services, infrastructure data, policy, gender, health outcome, epidemiology, risk prediction, risk

## Abstract

The collection of sexual orientation in routine data, generated either from contacts with health services or in infrastructure data resources designed and collected for policy and research, has improved substantially in the United Kingdom in the last decade. Inclusive measures of gender and transgender status are now also beginning to be collected. This viewpoint considers current data collections, and their strengths and limitations, including accessing data, sample size, measures of sexual orientation and gender, measures of health outcomes, and longitudinal follow-up. The available data are considered within both sociopolitical and biomedical models of health for individuals who are lesbian, gay, bisexual, transgender, queer, or of other identities including nonbinary (LGBTQ+). Although most individual data sets have some methodological limitations, when put together, there is now a real depth of routine data for LGBTQ+ health research. This paper aims to provide a framework for how these data can be used to improve health and health care outcomes. Four practical analysis approaches are introduced—descriptive epidemiology, risk prediction, intervention development, and impact evaluation—and are discussed as frameworks for translating data into research with the potential to improve health.

## Introduction

Research into health for individuals who are lesbian, gay, bisexual, transgender, queer, or of other identities including nonbinary (LGBTQ+) has consistently found that these populations experience poorer outcomes [1], with particularly strong and consistent evidence around poorer mental health for lesbian, gay, and bisexual adults [2]. LGBTQ+ health research typically uses 2 broad frameworks, sociopolitical or psychosocial models (where experiences of discrimination, victimization, stigma, and harassment are central to our understanding of health), and models of clinical, biomedical, and lifestyle risk factors [3]. Sexual health and HIV research falls mostly within biomedical frameworks; in practice, inequalities are interconnected [4,5]. Experiences of discrimination, harassment, and stigma have a profound impact on clinical health outcomes for LGBTQ+ communities [6]. Poorer health care access and quality among vulnerable groups exacerbate these impacts [7,8].

Data-driven LGBTQ+ health research has historically been based on convenience or purposive, rather than population-based samples [9], even in large-scale studies such as the National LGBT Survey in 2018 [1]. The collection of data on sexual orientation, gender, and transgender status has not been prioritized in routine sources [3]. Challenges in identifying understandable, meaningful, and acceptable measures, and concerns about LGBTQ+ respondents being able to answer questions safely, have been additional barriers to data collection [10].

However, data are improving [11]. The Equality Act in 2010 placed a statutory duty on public bodies in the United Kingdom to publish equality outcomes and report on progress in addressing disadvantage experiences by (among other characteristics) sexual orientation and gender reassignment [12], which had a strong positive impact. The Office for National Statistics has carried out development work on measures [13], and although the census in 2011 did not include questions about sexual orientation and transgender status, in part because of the concerns about respondent safety [10], by 2021 questions on both were included, with instruction statements that the questions were voluntary. In general, data collection for sexual orientation is more established than collection of data on transgender status and gender, although, for both, collections are improving.

This paper aims to provide a guide for how these improvements in routine data collection can potentially translate to improved health and health care outcomes.

## Pathways From Data to Improved Health

LGBTQ+ health research using routine data sits within wider health data science research frameworks which are designed to leverage person-level routine health data (collected either from routine contacts with services or from infrastructure data resources designed and collected for policy and research) to improve outcomes [14]. Analyses fall under four broad translational pathways: (1) descriptive epidemiology, (2) risk prediction, (3) informing innovation and improvement, and (4) impact evaluation.

These pathways have the potential to improve outcomes by (1) providing evidence to inform policy and practice (descriptive epidemiology), (2) better targeting of interventions and understanding of population health needs (risk prediction), (3) more rational health service developments (intervention development), and (4) information on effectiveness informing commissioning or funding decisions (impact evaluation), respectively.

Health data science as a field has struggled with equality, diversity, and inclusion [15] and there are problems across the whole discipline. Algorithmic biases in risk prediction models, including in how models are developed, with differentially poorer functioning for minoritized groups, or inequitable outcomes when the models are implemented, are currently a particular area of concern [16,17]. In addition, missing data contribute not only to poorer risk model development, but to a lack of basic descriptive epidemiology, informed intervention development, or equalities impact evaluation. For LGBTQ+ health research using routine data, the pathways to improved outcomes are the same, as are the challenges of missing data [18].

## Data

There are 5 groups or types of routine UK data sources—where information about sexual orientation and gender or transgender status are either well established or now starting to be recorded, beginning to address this lack of data. In the same way that LGBTQ+ health research balances both societal and biomedical models the data sources, which now include a collection of sexual orientation (more likely) or gender and transgender status (beginning to be introduced) reflect a balance of routine data from social and health sources. The five groups are (1) social science or societal data collections (including Understanding Society [19], birth cohort studies [20], educational cohort studies [21], and census data); (2) general and specific health surveys primarily designed to understand population health (including the Health Survey for England [22] and the National Survey of Sexual Attitudes and Lifestyles (NATSAL) [23]); (3) health services or patient surveys primarily designed for health service quality improvement (including the General Practice Patient Survey [24,25] and the Cancer Patient Experience Survey [26]); (4) health cohort studies (UK Biobank [27] and Our Future Health [28]); and (5) health records (including primary care research databases such as the Clinical Practice Research Datalink [29,30], and secondary and community services data sets, including the improving access to psychological therapies and mental health services data sets [31], and registry data, for example, cancer registry data [32]).

Put together, the data are starting to form a comprehensive collection but for each resource, there are strengths and limitations or challenges. For example, the Equality and Human Rights Commission was able to draw on quantitative evidence and data about sexual orientation and gender reassignment in work, education, and health in the State of the Nation report on equality and human rights in Britain published in November 2023 [33]. However, data access, sample size, measures of sexual orientation, gender, and health outcomes, and the ability to carry out longitudinal analysis and data quality vary across sources.

In terms of access to data, social science collections are primarily accessed without cost through the UK Data Archive; for sensitive fields, which often include sexual orientation or gender and transgender status, additional safeguards are in place. The UK Biobank and Our Future Health are 2 large biomedical data research cohorts accessed through trusted research environments (secure data hosting platforms) with relatively low but nonzero costs to researchers [28,34]. For all sources, access to data can require time and perseverance [11]. In terms of longitudinal follow-up, the UK Biobank is a mature cohort study, for which recruitment began in 2006 before sexual orientation and transgender status were routinely collected. Questions are included instead about sexual history, which provides some insight [34]. In contrast, Our Future Health for which recruitment began in 2022 has an inclusive gender question and questions about both sexual history and sexual orientation but only baseline data collection to date (recruitment is ongoing) [28].

Sample size is often a trade-off with detail. Understanding Society is a household panel survey designed to provide estimates about how life in the United Kingdom is changing and what stays the same over many years, with linked health and social data [19,35]. In common with other longitudinal and cohort data collections, the sample size is relatively small (about 40,000 people at baseline), compared, for example, to the General Practice Patient Survey which is a large cross-sectional survey designed to evaluate health care quality, which has a much larger sample size (about 700,000 responses) but with much less nuanced health and particularly, social measures recorded.

Pooling data across sources is an approach to increasing sample size [36], and again resources are improving, estimates across an in-depth range of health outcomes from the Health Survey for England using data from across 7 years have recently been published [22].

Data from electronic health records (EHRs), or data routinely recorded as part of clinical or health care encounters offer both detailed health outcome data and large sample size. The challenge is often that for EHR data collections, in contrast with research data infrastructure resources, or survey-based health data collections, measurement of sexual orientation and gender or transgender status is less good. In part, this is because these are resources not designed for research but primarily collected as clinical documentation. Pilots have begun to improve recording, to support audit and quality improvement. However, given both historic discrimination experienced by the LGBTQ+ community based on sexual orientation recording in medical records, and the interpersonal interlinking of recording and coming out to a care provider [37,38], this solution to data improvement is not simply a neutral administrative process. The reluctance of health care providers to ask about sexual orientation is a second barrier [39]. Recent research using EHR has provided insight by looking at transgender patients in primary care records based on prescribing and clinical codes, and this is an exciting area of progress [29,30]. These approaches have their own challenges, however, with historic clinical codes including outdated and discriminatory terminology still present in some older coded EHR records [18]. Legal barriers to identifying transgender patients after transition provide a further barrier to research using EHR; legislative changes have been required for recent quantitative analyses [40].

There are notable areas where data are poorer. Data governance and ethical challenges mean that data collections are much less likely to collect information on sexual orientation or gender from children. For example, some research studies have used proxies or less detailed response options where exact measures of ethnicity, gender identity, or disability cannot be asked [41]. Although HIV and sexual health research are well-studied topics in LGBTQ+ health research overall [42], routine data are usually more strongly safeguarded and less available for research, although measured in some collections.

## Applied Methodology

The improvement in data collection for LGBTQ+ health research in the last decade mean that the applied methodological research around the use of these data is also developing. Questions about the longitudinal consistency of self-reported sexual orientation and history have been explored; changes are more frequently reported at younger ages [34,43,44]. For sexual orientation, missing data have reduced over time since the question has been routinely introduced in surveys [45]. Secular trends are also being better understood [44,45], meaning that age, period, and cohort effects in LGBTQ+ health research can begin to be untangled [24]. Differential item functioning for new questions among groups for whom English is a second language is a current area of concern for new gender questions, although this is unlikely to be a methodological issue specific to these particular items. The challenges of longitudinal consistency in question wording needing to be balanced against requirements for relevant and up-to-date survey items is again a methodological challenge not specifically limited to questions about sexual orientation and gender. New, nuanced, tools for understanding gender are beginning to be developed [46]; however, space constraints in surveys mean that often only single items are asked. While free text or more in-depth response options (or allowing multiple rather than single responses) are more inclusive [47,48], these nuanced data are often excluded from quantitative reporting. Data for people who identify as asexual are very limited, as are data for people with variations in sexual characteristics.

## How Have These Data Translated Into Applied Research?

As LGBTQ+ routine data are improving, the insights that come from descriptive epidemiological LGBTQ+ health research are also developing. For example, historically, studies using routine data have been able to consider cancer risk factors such as, smoking and alcohol consumption [49-51], more easily than rarer cancer outcomes such as incidence. Limited sample size and poorer measurement of outcomes mean that earlier studies looking at cancer were cross-sectional and could only consider cancer prevalence without disaggregation by diagnosis [52]. Larger cross-sectional data sets have allowed disaggregation of diagnoses among lesbian, gay, and bisexual patients with cancer, identifying disparities primarily in HIV and human papillomavirus–associated cancers [26]. More recent work has for the first time in the United Kingdom been able to look at the impact of higher smoking prevalence identified in earlier studies on lung cancer incidence, using the UK Biobank resource [53], connecting both biomedical and sociopolitical frameworks; the LGBTQ+ community has historically been targeted by tobacco marketing.

Inequalities in LGBTQ+ mental health outcomes have also been well established through a series of studies and meta-analyses using routine data from the United Kingdom [54]. In our recent work collaboratively exploring LGBTQ+ research priorities, intersectionality (understanding the interdependent and overlapping systems of discrimination and disadvantage) was identified as an area of research need; and race, ethnicity, and socioeconomic inequalities were particularly highlighted [55]. Larger sample sizes mean that intersectionality can now begin to be explored quantitatively [56]; newer longitudinal collections are providing additional insight [57].

Again, it is not just biomedical models that are important. Routine educational data sets have been important in highlighting the higher levels of bullying experienced by young LGBTQ+ people in schools [21].

However, we also know that on its own research describing inequalities experienced by LGBTQ+ adults will not lead directly to improved outcomes. Process measures of care quality are often easier to improve than more tangible health outcomes. However, although disparities in primary care access, communication, and satisfaction were measured routinely between 2011 and 2017, inequalities experienced by lesbian, gay, and bisexual adults persisted across the time period [58].

Impact evaluation is a second pathway, therefore, where routine data are beginning to be used to provide insight with the potential to change the care process and improve LGBTQ+ health outcomes. The collection of sexual orientation information in the Improving Access to Psychological Therapies data set has allowed inequalities evaluation of these services for lesbian, gay, and bisexual adults, finding that they were not as effective as for heterosexual service users [31]. In contrast, an inequalities evaluation of the introduction of telephone triage in general practices using the GP Patient Survey found that although there was variation between practices in outcomes, for different groups of patients within the same practice, including lesbian, gay, and bisexual adults, there was no evidence of differential impact on access to primary care [59]. The Millennium Cohort Study has been used to understand the differential impact of the COVID-19 pandemic on sexual minority groups [57], as has Understanding Society [60].

## Where to Next—Routine Data Analysis?

Routine data for LGBTQ+ health research are much better today, in 2024, than they were even 10 years ago. Sexual orientation has now been collected in many sources for over a decade and more diverse and inclusive gender measures are being introduced, and are established in some collections. Of course, measurement needs to continue and is continuing to improve, and there are limitations and barriers; no data set alone is perfect. However, across the spectrum of sources, there is a real depth of data now available and in terms of research, the data are good enough now to at least start thinking properly about how we can use these resources to improve LGBTQ+ health and tackle inequalities.

In terms of data development, of course linkage is 1 exciting potential future avenue, with the linked 2011 census and routine health care data in Scotland providing a possible model for future development. But in reality, using routine data for LGBTQ+ health research lies within the wider UK research landscape for using routine data overall. Here the Goldacre review probably shines some light on the direction of travel [61]. Access is becoming more cautious, and data are becoming more securely safeguarded, new frameworks and solutions are needed to ensure that access continues and barriers do not increase [62]. For sensitive fields such as sexual orientation, gender, and transgender status, this is particularly important, but it is likely that time, patience, and perseverance are going to continue to be required when working in this space. As a balance to concerns about the use of person-level data, tools sharing aggregate data such as the census resources from the United Kingdom’s Office for National Statistics [63], and the analysis tool for the GP Patient Survey remain important resources and provide real insight.

So, the question remains, how are we going to use these data to improve LGBTQ+ health? Although the data are better, the approaches have not changed and the methodological answers to the pathways from data to improved health remain the same. The four pathways are (1) descriptive epidemiology, (2) risk prediction, (3) informing innovation and improvement, and (4) impact evaluation.

Given the recentness of the data improvements and that data resources are still improving, there remains a real need for basic epidemiological descriptive work using these new data to answer questions and provide insight where simply the data have not been available before. More in-depth analyses, analyses considering longitudinal changes, and better measures of health and health outcomes, as well as sexual orientation and gender and transgender status, are all part of this. Frameworks for addressing health inequalities require researchers to go beyond simply describing known inequities [14], but for LGBTQ+ health there is still an evidence gap where descriptive epidemiology that focuses on areas where research could have an impact on policy has a place.

Maybe the results will be unsurprising, and research may show that inequalities have not disappeared as the data have improved, but the work is still important, and insight is still needed.

Risk prediction as a field has real challenges ahead to get to grips with equality, diversity, and inclusion, and this needs to include LGBTQ+ health. For transgender health specifically, there are some more questions to ask around risk model development; the exclusion of transgender adults from the development of some risk scores [47], and lack of clarity about how to implement scores based on binary gender or sex classifications are some specific issues to add to these [18]. Although methodological work is still needed to understand the best way to develop and implement risk scoring for transgender patients to avoid potentially both under- and overtreatment, the critical first step is to ensure that data used for model development do not exclude transgender populations before the research begins.

In terms of intervention development and audit, the improvement of data is important to ensure that evidence-based interventions are developed and part of wider thinking about how routine data can improve health and LGBTQ+ health in particular. Specific clinical data sets, such as cancer data collections or more in-depth surveys such as NATSAL will be particularly important in this domain. Much local evaluation of LGBTQ+ health interventions remains qualitative [64], and the evidence base for health equity audits to address inequalities remains poor [65].

The importance of including explicit inequality analyses in impact evaluations remains a key analysis strategy for improving health. Even when interventions are not LGBTQ+ specific, there may or may not be an inequitable impact. This kind of routine equalities impact work for LGBTQ+ and other groups is central to the drive the Equality Act has given to the improvement in data that we have seen, and needs to become a routine part of evaluative work.

## Where to Next—LGBTQ+ Health Research?

The data are good enough now for routine data to play a substantive part in LGBTQ+ health research, and there are clear and realistic pathways for how this research can potentially improve health. This comes within the wider context of flourishing LGBTQ+ health research overall [66]. Health and health care are complex [67]. It is not a linear pathway from data to improved health outcomes; but good research can play a part.

There are particular challenges for health research with LGBTQ+ children and young people, where data are often less frequently collected and ethical and governance considerations are particularly important, and there is an identified need for more research [68,69].

The co-option of research findings into homophobic or transphobic narratives is a further difficult area, as are avoiding some of the blind spots around equality, diversity, and inclusion in routine data research that are beginning to be identified particularly in risk prediction work [15]. Good communication and cautious interpretations of findings are part of the solution, as are patient and public involvement, and the involvement of LGBTQ+ communities in identifying research priorities and in carrying out research [55]. Best practice guidance for LGBTQ+ health research [70], inclusive public involvement [71], and involvement in LGBTQ+ health research [72] provide some signposts for researchers.

## Conclusions

Descriptive epidemiology, risk prediction, informing innovation and improvement, and impact evaluation are 4 practical pathways from data to improved health. Data for LGBTQ+ health research are now good enough and improving. We know that health inequalities exist, within both societal and biomedical frameworks. Research with strong public involvement, good clear communication, and stakeholder involvement is key, as in all research. Overall, this is a positive story for routine data. We are at the stage where the analysis of routine data can contribute to making real practical steps toward informing policy and practice, better targeting of interventions and understanding of population health needs, more rational health service developments, informing commissioning or funding decisions, and improving LGBTQ+ health.

## Abbreviations

EHR: electronic health record
LGBTQ+: lesbian, gay, bisexual, transgender, queer, or other identities including nonbinary
NATSAL: National Survey of Sexual Attitudes and Lifestyles
